# Influenza Vaccine Induces Intracellular Immune Memory of Human NK Cells

**DOI:** 10.1371/journal.pone.0121258

**Published:** 2015-03-17

**Authors:** Yaling Dou, Binqing Fu, Rui Sun, Wenting Li, Wanfu Hu, Zhigang Tian, Haiming Wei

**Affiliations:** 1 Institute of Immunology, School of Life Sciences and Hefei National Laboratory for Physical Sciences at the Microscale, University of Science and Technology of China, Hefei, China; 2 Anhui Provincial Center for Disease Control and Prevention, Hefei, China; University of Georgia, UNITED STATES

## Abstract

Influenza vaccines elicit antigen-specific antibodies and immune memory to protect humans from infection with drift variants. However, what supports or limits vaccine efficacy and duration is unclear. Here, we vaccinated healthy volunteers with annual vaccine formulations and investigated the dynamics of T cell, natural killer (NK) cell and antibody responses upon restimulation with heterologous or homologous influenza virus strains. Influenza vaccines induced potential memory NK cells with increased antigen-specific recall IFN-γ responses during the first 6 months. In the absence of significant changes in other NK cell markers (CD45RO, NKp44, CXCR6, CD57, NKG2C, CCR7, CD62L and CD27), influenza vaccines induced memory NK cells with the distinct feature of intracellular NKp46 expression. Indeed, surface NKp46 was internalized, and the dynamic increase in NKp46(intracellular)^+^CD56^dim^ NK cells positively correlated with increased IFN-γ production to influenza virus restimulation after vaccination. In addition, anti-NKp46 antibodies blocked IFN-γ responses. These findings provide insights into a novel mechanism underlying vaccine-induced immunity and NK-related diseases, which may help to design persisting and universal vaccines in the future.

## Introduction

Flu viruses mutate easily, especially the influenza A hemagglutinin (HA) and neuraminidase (NA) antigens. This antigenic drift/shift that occurs in flu viruses, including H1N1 (2009, California) and H7N9 (2013, China) [1], has caused worldwide pandemics and poses a threat to human health. Although seasonal influenza vaccines effectively prevent flu infection and outbreaks during a particular season, vaccination cannot provide long-term protection, and humans can still suffer from the flu after vaccination [2].

Currently, vaccines are developed empirically; the WHO Global Influenza Surveillance Network recommends strains (one influenza A H1N1, one influenza A H3N2 and one influenza B virus) for vaccination before each annual epidemic. Little is known about how vaccines activate immunity and what restricts immune persistence. Long-term protection requires two factors: antibody persistence and immune memory. Neutralizing antibodies have limitations, as circulating strains are unlikely to harbor vaccine-derived antigens [3]. On the other hand, although T cells are thought to play a pivotal role in vaccine efficacy, the most potent CD8 T cell—inducing influenza vaccine does not induce sufficient cross-reactive CD8 T cells to provide substantial protection against lethal non-homologous influenza A virus challenge [4,5]. Besides B-cell and T-cell responses, an advantage of natural killer (NK) cell responses may be to provide broader immunity to multiple influenza virus subtypes; indeed, it was reported that influenza infection caused a significant increase in NK cell activity in human volunteers experimentally challenged with a wild-type influenza virus [6,7]. Protective effects of NK cells against viral infections may be mediated by cytokines such as IFN-γ, an antiviral cytokine that contributes to inhibiting viral replication and eliminating the virus from the host [8]. Several studies on human NK cells showed that NK cells can increase their IFN-γ response to influenza antigen [6,9], suggesting that NK cells may play an important role in controlling flu infection. Thus far, however, there remains a lack of longitudinal studies on human NK cell responses to influenza virus vaccines.

Immune memory forms the basis of vaccination. Memory cells are long-lived and respond rapidly against the same pathogen in subsequent infections. Besides B and T cells, NK cells also bear adaptive features [10]. NK memory was previously shown in 3 models: hapten-induced contact hypersensitivity (CHS) [11,12], mouse cytomegalovirus (MCMV)-induced antigen-specific memory [13,14] and cytokine-induced memory [15,16]. In the antigen-specific MCMV model, the Ly49H—m157 interaction expanded Ly49H^+^ NK cells and elevated their cytotoxic and IFN-γ responses [13]. Several NK receptors are specific for viral ligands; among these, NKp46 is an activating receptor that directly recognizes influenza-encoded HA and mediates cytolysis of infected cells [17–19]. In humans, prolonged persistence of enhanced IFN-γ was observed in human cytomegalovirus (HCMV), hantavirus and herpes simplex virus (HSV-2) infections [20–22]. However, the molecular mechanisms underlying NK memory and persistence remain unclear, and receptor interactions with viral ligands may help to better understand NK memory.

## Materials and Methods

### Ethics statement, study subjects and vaccination

We enrolled 27 healthy adult volunteers ranging from 20 to 47 years of age (mean = 26 y, S1 Table). No volunteer had previously received influenza virus vaccination. The study was approved by the Ethical Committees of the University of Science and Technology of China (Approval number: USTCEC201300001) and was performed in accordance with the Declaration of Helsinki. Written informed consent was obtained from all volunteers before enrollment.

To ensure that the volunteers were relatively naive to the strains used for restimulation, we measured plasma influenza antibody IgM levels by ELISA and randomly selected 13 IgM-negative individuals for further study. Eleven of the 13 subjects received an inactivated split influenza vaccine composition (FluaRIX, GlaxoSmithKline) by intramuscular (i.m.) injection on day 0 at the Hefei Center for Disease Control and Prevention. Six were immunized with the 2010–2011 vaccine composition (subjects #1–6), 2 were immunized with the 2012–2013 vaccine composition (subjects #9, #10) and 3 were immunized with the 2013–2014 vaccine composition (subjects #11–13). The 2010–2011 vaccine contained A/California (H1N1)-like, A/Perth (H3N2)-like and B/Brisbane-like strains. The 2012–2013 and 2013–2014 vaccines shared the A/California (H1N1)-like and A/Victoria (H3N2)-like strains, but differed in their influenza B strains (2012–2013: B/Wisconsin/1/2010-like; 2013–2014: B/Massachusetts-like). Two subjects (subjects #7, #8) served as unvaccinated controls.

### PBMC preparation

For vaccinated subjects (S1 Table, subjects #1–6, #9–13), we collected whole venous blood on day 0 (prior to vaccination) and at approximately 1-month intervals following vaccination for up to 6 months. Samples from the unvaccinated control subjects (subjects #7, #8) were collected at the same intervals after the first collection (day 0). Plasma obtained from different donors at various time points were stored at -80°C. Fresh human peripheral blood mononuclear cells (PBMCs) were isolated by Ficoll-Hypaque (Solarbio, Beijing, China) density-gradient centrifugation. In some experiments, NK cells from subjects #9–13 were purified to >90% using the human NK cell isolation kit (Miltenyi Biotec, Bergisch Gladbach, Germany). PBMCs and NK cells were then prepared for in vitro stimulation or staining.

### Virus preparation

Influenza A/PR/8/34 strain (H1N1) virus was injected in the allantoic cavity of 10-day-old specific pathogen—free embryonated chicken eggs (Merial-Vital Laboratory Animal Technology Co. Ltd., Beijing, China). Virus-containing allantoic fluids were harvested 72 hours after inoculation, and HA titers were tested using an HA assay with fresh chicken red blood cells. In some experiments, virus was inactivated by incubation in a 56°C water bath for 35 minutes before being used for stimulation. All viruses were stored at -80°C. Human respiratory syncytial virus (RSV) Long strain A were grown in Hep-2 cells and assayed for infectivity. RSV stock was frozen at -80°C at a 1 × 10^7^ PFU/mL concentration. The A/California/7/2009 (H1N1) strain shared among the 3 vaccines was kindly provided by Prof. Bing Sun (Institute Pasteur of Shanghai, Chinese Academy of Sciences, Shanghai, China). Influenza virus A/H1N1, A/H3N2 and B strains were kindly provided by Jun He (Anhui Provincial Center for Disease Control and Prevention, Hefei, China).

### Plasma influenza A virus antibody assay

Plasma influenza A virus IgM antibody titers were assayed using the influenza A virus IgM ELISA Kit (MBS494222, MyBioSource, San Diego, CA) according to the manufacturer’s protocol.

### HI assay

HA-specific antibody titers to influenza virus A/H1N1, A/H3N2 and B strains were determined at day 0, 2 months and 6 months post-vaccination by the hemagglutination-inhibition (HI) assay. Briefly, collected plasma was first treated with a receptor-destroying enzyme (RDE, cholera filtrate) at 37°C overnight to remove non-specific HA inhibitors. The RDE reaction was then stopped by heating the samples at 56°C for 30 minutes. Samples were diluted from 1:2 to 1:2048, and HI titers were defined as the highest dilution rate that caused complete hemagglutination inhibition against 4 hemagglutinating units of virus.

### Flow cytometric analysis

PBMCs were incubated with 5% normal mouse serum to block FcRs and then stained with the following mAbs: APC-Cy7–anti-CD3, PE-Cy7–anti-CD56, Alexa 488–anti-CD56, FITC—anti-CD45RA, FITC—anti-CD57, FITC—anti-CD62L, PE—anti-CD57, PE—anti-NKp44, PerCP-Cy5.5–anti-CD45RO, PerCP-Cy5.5–anti-CD27, APC—anti-NKp46, APC—anti-CXCR6 and PE-Cy7–anti-CCR7; all antibodies and isotype-matched controls were purchased from BD Biosciences (San Jose, CA). After incubating for 30 minutes at 4°C, samples were washed and resuspended in PBS with 1% paraformaldehyde. Data for the stained cells were acquired on a flow cytometer (Becton Dickinson, Franklin Lakes, NJ) and analyzed using FlowJo software 7.6.1 (TreeStar, Inc., Ashland, OR).

### Detection of surface and intracellular NKp46

For detecting surface NKp46 expression, cells were incubated with normal mouse serum and then stained with PE—anti-NKp46 antibody or isotype control mouse IgG1, κ at 4°C for 30 minutes. Samples were then washed and resuspended in PBS with 1% paraformaldehyde before FACS analysis. For detecting intracellular NKp46 expression, cells were first surface-stained with anti-CD3 and anti-CD56 antibodies, fixed with 3% paraformaldehyde and permeabilized with 0.5% saponin. After washing, cells were incubated with normal mouse serum in the presence of 0.5% saponin for 30 minutes and then stained intracellularly with PE—anti-NKp46 in the presence of 0.5% saponin; for control cells, isotype control antibody was added and incubated for 30 minutes at 4°C. Cells were then extensively washed in 0.5% saponin buffer and PBS before FACS analysis.

### Ex vivo stimulation and detection of IFN-γ production

PBMCs or purified NK cells were resuspended at 4 × 10^6^/mL with complete RPMI 1640 medium (consisting of 10% FBS, 100 U/mL penicillin and 100 U/mL streptomycin) and incubated with or without 2000 HA/mL inactivated A/PR8 virus in 96-well U-bottom plates for 18 hours. In some experiments, cells were also stimulated with different influenza virus subtypes, or cells were infected with RSV at a multiplicity of infection (MOI) of 1. For the last 4 hours, 1.77 μg/mL monensin (Sigma, St. Louis, MO) was added to all cultures. Cells were harvested, stained with surface markers as previously described, washed, fixed and permeabilized with 0.5% saponin. They were then stained with FITC-conjugated anti—IFN-γ mAb (BD Biosciences), washed and resuspended in PBS with 1% paraformaldehyde. Data were acquired using a flow cytometer.

In some experiments, cells were co-cultured with 10 μg/mL anti-NKp46 blocking antibodies (BD Biosciences) on ice for 1 hour. Cells were then stimulated for 18 hours with 2000 HA/mL A/PR8 or 3 μg/mL FluaRIX vaccine. A neutralizing rat anti—human IL-2 antibody (4 μg/mL) (BD Biosciences) was used to neutralize IL-2 in culture. The corresponding mouse IgG1, κ isotype and rat IgG2a, κ isotype antibodies were used as the respective controls for the anti-NKp46 and anti—IL-2 antibodies.

### Confocal fluorescence microscopy

Purified NK cells obtained at each time point during the study were attached to poly-D-lysine—treated slides, fixed for 20 minutes at room temperature with 3.7% (volume/volume) paraformaldehyde and permeabilized with 0.1% Triton X-100 in PBS-1% (weight/volume) BSA for 10 minutes. After blocking for 30 minutes with donkey serum at room temperature, cells were stained with goat anti—human NKp46 (Santa Cruz Biotechnology, Santa Cruz, CA) overnight at 4°C. The slides were then washed in PBST (PBS with 0.05% Tween 20) for 5 minutes, followed by washing 3× in PBS (5 min/wash). Secondary antibodies (FITC-conjugated donkey antibodies specific for goat immunoglobulin) were added for 1 hour in the dark at room temperature. Nuclei were stained with DAPI (Santa Cruz Biotechnology) for 1 minute. Coverslips were washed, mounted in ProLong Gold antifade reagent (Invitrogen, Carlsbad, CA) and examined with an LSM 710 laser-scanning microscope (Carl Zeiss, Jena, Germany). A Plan-Apochromat 63× (1.40 NA) oil-immersion objective (Carl Zeiss) was used for image acquisition. Images were analyzed using ZEN 2009 software (Carl Zeiss).

### Statistical analysis

Data analyses were performed using GraphPad Prism 5 (San Diego, CA). To determine statistical significance, a two-tailed Student’s *t*-test was used to compare 2 groups. One-way ANOVA was used to compare significant differences among 3 or more groups, followed by a Bonferroni multiple comparison test, or a Student’s *t*-test. Statistical significance was defined as *P* values < 0.05: *P < 0.05, **P < 0.01, ***P < 0.005.

## Results

### Influenza vaccination increases IFN-γ production by human NK cells

We established an influenza vaccine model in healthy adults (S1 Fig.). Eleven of these 13 volunteers (subjects #1–6, #9–13) received an inactivated split influenza vaccine, and 2 were not inoculated for use as unvaccinated controls (subjects #7, #8).

**Fig 1:**
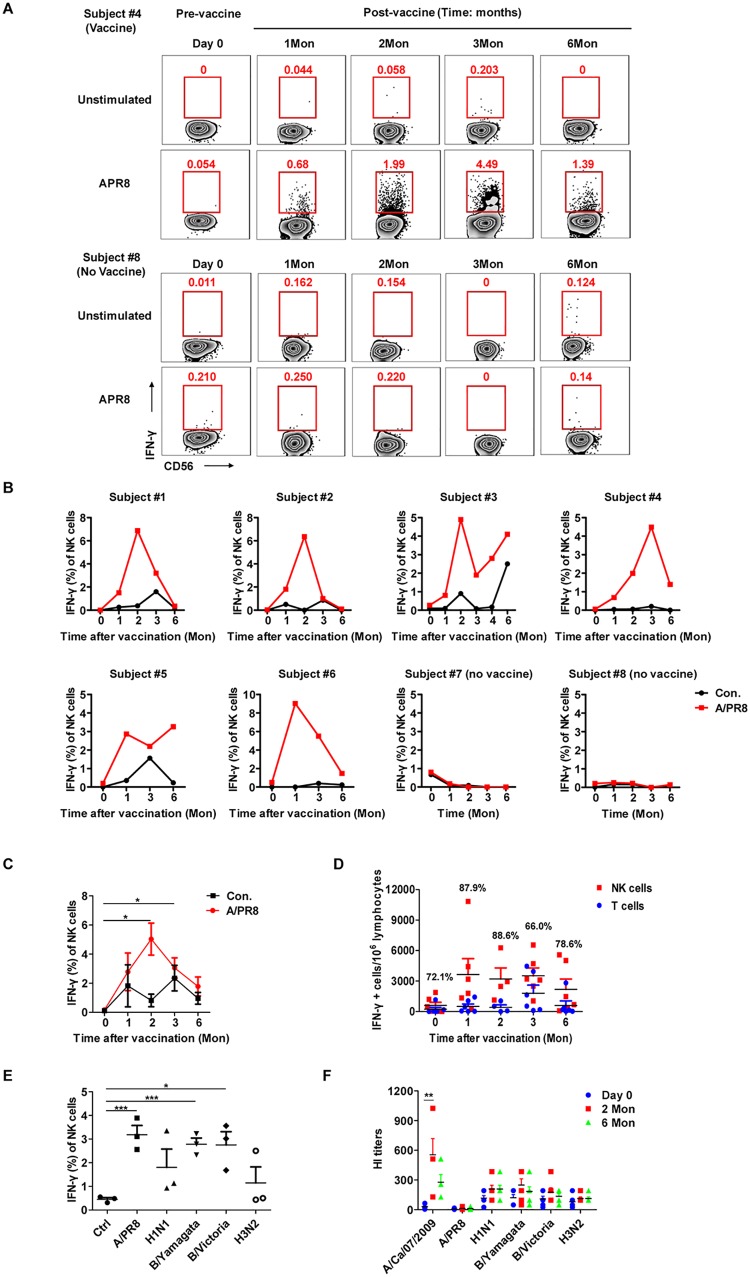
Dynamic IFN-γ responses from human NK cells to heterologous influenza virus restimulation. (**A**) Fresh PBMCs from vaccinated (subject #4) and unvaccinated (subject #8) donors were cultured with or without inactivated influenza A/PR8 at the indicated time points. Intracellular IFN-γ production from gated CD3^−^CD56^+^ human NK cells was detected 18 hours later by FACS. (**B**) Long-term monitoring of NK cell—derived IFN-γ from vaccinated (subjects #1–6) and unvaccinated (subjects #7, #8) donors. Plots represent NK cells from individual donors against A/PR8 (red) or unstimulated control (black) at each time point. (**C**) IFN-γ^+^ NK cell percentage detected before and after vaccination (n = 6). (**D**) Absolute IFN-γ^+^ cell number from NK (red) or CD3^+^ T (blue) cells to the total number of IFN-γ^+^ cells responding to A/PR8 at different time points. Numbers above each column represent the mean proportion of IFN-γ^+^ NK cells (n = 6). (**E**) Fresh PBMCs from subjects 2 months after vaccination were cultured with or without (control) the corresponding influenza virus for 18 hours, and intracellular IFN-γ production on gated CD3^+^CD56^−^ human NK cells was detected by flow cytometry (n = 3). (**F**) Plasma was obtained from vaccinated subjects before (day 0, blue) and after (2 months, red; 6 months, green) vaccination, and the influenza-specific antibodies were investigated by the hemagglutination inhibition (HI) assay (n = 6). The data in C, D, E and F are presented as the mean ± SEM. *P < 0.05, **P < 0.01, ***P < 0.005; Student’s *t*-test.

Memory cells are long-lived and exhibit rapid recall responses, including robust cytokine production. To determine whether human NK cells could develop influenza-specific memory, IFN-γ production in response to inactivated A/PR8 (as a heterologous recall antigen) by CD3^−^CD56^+^ NK cells in PBMCs was evaluated over time ex vivo. Expectedly, A/PR8 stimulation highly upregulated CD3^−^CD56^+^ NK cell IFN-γ secretion in samples from vaccinated individuals compared to unstimulated or pre-vaccination controls (Fig. 1A and B), and IFN-γ remained low with or without A/PR8 stimulation in unvaccinated controls. In contrast, although the frequency and peak of IFN-γ–producing cells differed for each vaccinated donor (50% [3/6] peaked at 2 months; subject #4 peaked at 3 months, and IFN-γ was still detected at 6 months), robust NK cell recall responses were observed at 1 month and were sustained for at least 6 months. Statistical analysis showed that NK cell—derived IFN-γ production to A/PR8 gradually decreased after the 2-month peak, returning to normal after 6 months (Fig. 1C).

Total IFN-γ–producing cells greatly increased after vaccination, most dramatically from NK cells (Fig. 1D), which represented 60–90% of all A/PR8-responding IFN-γ^+^ cells throughout the study. Since IFN-γ–producing CD3^+^ T cells from vaccinated subjects did not substantially increase upon A/PR8 restimulation, NK cells rather than T cells comprised the most significant recall effector-cell population after vaccination.

We then hypothesized that the observed increase in IFN-γ responses from NK cells might provide immunity to a broader range of influenza virus subtypes. To determine this, we stimulated PBMCs 2 months after vaccination with different influenza virus subtypes, including A/PR8, A/H1N1, A/H3N2 and 2 B strains, and then detected IFN-γ from NK cells (Fig. 1E). We found that various influenza subtypes were able to elicit IFN-γ from NK cells upon ex vivo stimulation, including subtypes in the seasonal vaccines (like B strains) as well as those not in the seasonal vaccines (like A/PR8), suggesting that NK cells could provide broad immunity against different influenza subtypes. In contrast to the NK recall responses, we surprisingly found that antibody responses against various influenza subtypes were not as broad. As assessed by the influenza HI assay, increased HI titers were only detected to A/California/7/2009 (H1N1) antigens, which were shared among the seasonal vaccines, but were not detected against antigens from heterologous viruses not included in the seasonal vaccines (like A/PR8) (Fig. 1F).

The aforementioned results indicate that human NK cells produce increased and sustained IFN-γ recall responses to multiple influenza subtypes after receiving the seasonal influenza vaccine. This is in contrast to the elicited antibody response, which was effective to antigens within the vaccine strain but afforded little protection against heterologous strains like the A/PR8 virus. Thus, these results imply that an advantage of the NK cell memory-like response may be its ability to provide broad immunity against many influenza subtypes, even though this enhancement persists for only 6 months after vaccination. Given that the known protective role of IFN-γ during the recall response to influenza A virus is to clear the virus from mouse hosts [8], NK cells may play an important role early (~6 months) after vaccination to provide vaccine-induced cross-protection.

### Influenza vaccination downregulates membrane surface NKp46 expression on CD3^−^CD56^dim^ NK cells

**Fig 2:**
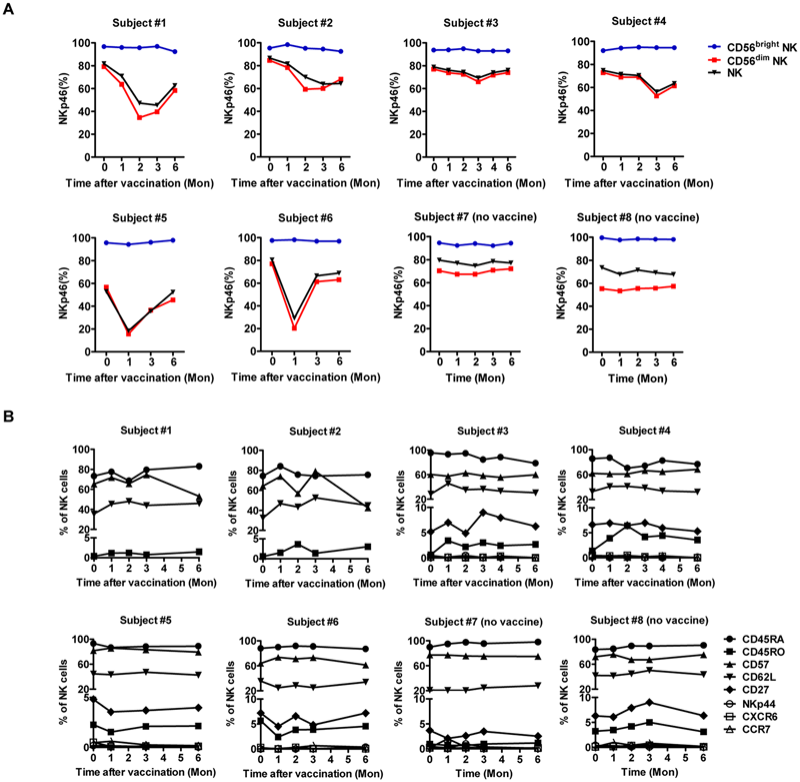
Kinetics of surface NKp46 expression after influenza vaccination. (**A**) FACS analysis of surface NKp46^+^ NK cell percentages in total NK (black), CD56^bright^ NK (blue) and CD56^dim^ NK (red) cells from 8 donor PBMCs at the indicated time points. (**B**) Longitudinal and phenotypic analysis of NK cells. (CD27^+^, NKp44^+^, CXCR6^+^ and CCR7^+^ NK cell frequencies for subjects #1 and #2 were unavailable.)

Particular receptors are associated with memory NK cells in mice. Ly49H, which specifically binds to viral m157, mediates profound NK expansion to MCMV [13]. In the classic CHS model [11], DX5^-^Ly49A^+^ NK cells represent liver-resident memory NK cells [23,24]. To identify candidate activating receptors that might recognize influenza during vaccination, NK surface molecules were examined in both vaccinated and control subjects. We initially focused on NKp46, the activating receptor specific for influenza A—encoded HA. NKp46 was significantly downregulated 1 month post-vaccination (Fig. 2A). NKp46^+^ NK cell percentage was lowest at 3 months (the absolute lowest occurred in subject #5 at 1 month) but gradually normalized over 6 months. Furthermore, while NKp46^+^CD56^bright^ NK cell frequency remained relatively stable (mean: 90%; range: 78.6–97.2%) in all subjects, NKp46^+^CD56^dim^ NK cell frequency significantly decreased in vaccinated subjects (the greatest decrease occurred in subject #6, from 80% [day 0] to 20% [1 month]).

We also evaluated other memory-associated surface molecules on total peripheral CD3^−^CD56^+^ NK cells by FACS (Fig. 2B). CD45RO and CD45RA expression distinguishes memory from naive T cells, and CD45RO serves as an NK memory marker during *Mycobacterium tuberculosis* infection [25]. Most peripheral NK cells expressed CD45RA (~90%), whereas <5% expressed CD45RO; both percentages remained stable following vaccination. NKp44 also potentially recognizes HA [18], and CXCR6 (CD186) is critical for antigen-specific NK memory for haptens and viruses in the mouse model [12]; however, NKp44, CXCR6 and CCR7 [26] expression on peripheral NK cells was not detected. CD57 and NKG2C are NK maturation markers implicated in NK expansion and memory during human hantavirus and HCMV infection [20,27], where CD57^+^NKG2C^hi^ NK cells selectively expand during acute HCMV infection [21]. However, CD57 and NKG2C expression did not change, and no CD57^+^NKG2C^hi^ NK subsets were found (S2 Fig.). CD27 and CD62L were associated with memory and activation [28,29], but CD62L^+^ and CD27^+^ NK cell frequency remained stable following vaccination in most subjects. Besides NKp46, no differences were observed in expression of the above receptors between the vaccinated and unvaccinated subjects. Taken together, influenza vaccination uniquely downregulated surface NKp46 on CD3^−^CD56^dim^ NK cells within 1 month, which returned to pre-vaccination levels during the latter 3 months of the study.

### Intracellular NKp46 expression correlates with increased IFN-γ in response to influenza virus restimulation

**Fig 3:**
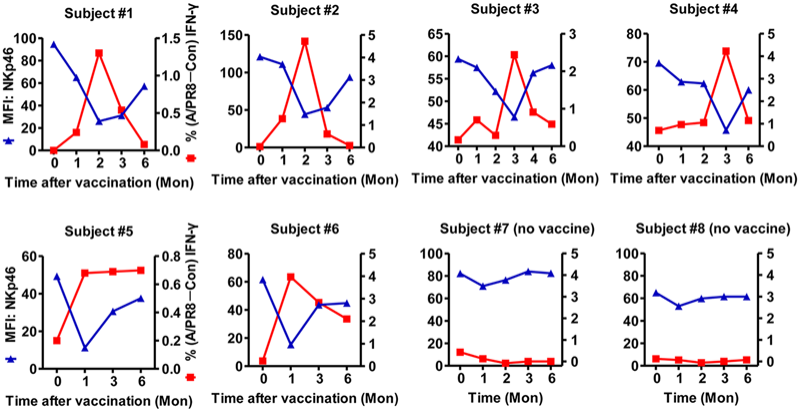
NKp46(S) expression on CD56^dim^ NK cells after vaccination inversely correlates with IFN-γ secretion to A/PR8 restimulation. FACS analysis of NKp46 expression (MFI) on CD3^−^CD56^dim^ NK cells from PBMCs of both vaccinated and control subjects (left y-axis, blue). PBMCs restimulated ex vivo with or without A/PR8 were evaluated at 18 hours for IFN-γ secretion by gating on CD3^−^CD56^dim^ NK cells (right y-axis, red). NK responsiveness was calculated as: % A/PR8-stimulated IFN-γ^+^CD3^−^CD56^dim^ NK cells—% control IFN-γ^+^CD3^−^CD56^dim^ NK cells.

The kinetic NKp46 changes that occur upon vaccination may relate to the observed increase in IFN-γ by NK cells. Upon further analysis, we found that low NKp46 expression remarkably accompanied high IFN-γ secretion from NK cells in response to A/PR8 restimulation in most vaccinated subjects (Fig. 3). In contrast, NKp46 expression remained stable with low IFN-γ in CD56^dim^ NK cells from unvaccinated controls (subjects #7, #8). Indeed, IFN-γ production to A/PR8 restimulation only increased during periods of reduced NKp46 and gradually weakened upon NKp46 restoration; no IFN-γ was detected at 6 months when NKp46 returned to pre-vaccination levels.

**Fig 4:**
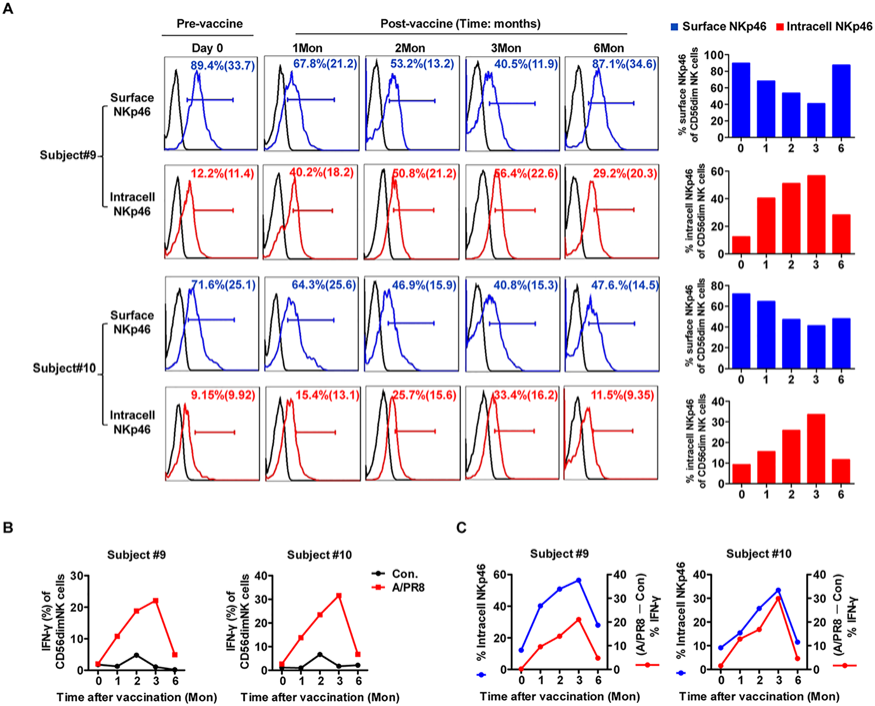
NKp46(I) expression associates with increased IFN-γ secretion to influenza virus restimulation. (**A**) FACS analysis of surface and intracellular NKp46 expression from pre- and post-vaccination PBMCs (subjects #9, #10). Histograms show percentage and MFI of NKp46(S) (red), NKp46(I) (blue) or isotype controls (black) on CD3^−^CD56^dim^ NK cells. (**B**) Plots represent dynamic IFN-γ production from CD56^dim^ NK cells to A/PR8 restimulation (red) or unstimulated control (black). (**C**) Dynamics of intracellular NKp46 proportions (left y-axis, blue) and NK responsiveness (as calculated in Fig. 4; right y-axis, red) on gated CD3^−^CD56^dim^ NK cells.

We next determined whether receptor internalization downregulated surface NKp46 expression after NK cells interacted with influenza-expressed HA, as previously reported [30,31]. We gated on CD3^−^CD56^dim^ NK cells within the lymphocyte gate of PBMCs to assess surface and intracellular NKp46 expression (S3 Fig.). Consistent with the above results, surface NKp46 was significantly downregulated on CD56^dim^ NK cells from fresh PBMCs at the indicated time points (Fig. 4A). However, vaccination increased the frequency of CD56^dim^ NK cells expressing intracellular NKp46, peaking at 3 months in subjects #9 (56.4%) and #10 (33.4%); notably, NKp46(intracellular)^+^CD56^dim^ NK cell frequency reduced to 29.2% and 11.5%, respectively, at 6 months, similar to baseline levels (day 0: 12.2% and 9.15%, respectively).

While surface NKp46–positive (NKp46[S]^+^) NK cell frequency decreased, intracellular NKp46–positive (NKp46[I]^+^) NK cells increased. Pre-vaccination, CD56^dim^ NK cells exhibited little intracellular NKp46 and no IFN-γ in response to A/PR8. In contrast, after vaccination, CD56^dim^ NK cells upregulated IFN-γ in response to A/PR8 restimulation, which remained low in the absence of A/PR8 (Fig. 4B). NKp46(I)^+^CD56^dim^ NK cells increased within 1 month, peaked at 3 months and then dropped to near baseline levels by 6 months (Fig. 4C), coinciding with the wave of IFN-γ production against A/PR8. Taken together, the dynamic increase in NKp46(I)^+^CD56^dim^ NK cells positively correlated with increased IFN-γ production to A/PR8 restimulation after vaccination, suggesting a close association between these two events.

**Fig 5:**
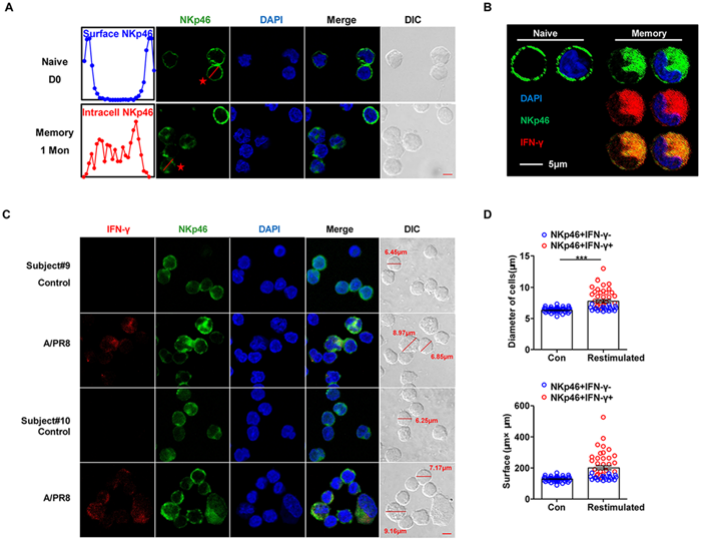
Memory NK cell phenotypes and secondary-response features. (**A**) NK cells from vaccinated-donor PBMCs were purified at day 0 and then again at 1 month post-vaccination for immunofluorescence assays. Histograms (left) show NKp46 fluorescence intensity on the red lines of cells marked with an asterisk (red). Graphs show NKp46(I) in memory NK cells (1 month post-vaccination) versus NKp46(S) on naive NK cells (day 0). (**B**) Representative confocal micrographs show differences between naive (day 0) and memory (3 months post-vaccination) cells in terms of NKp46 (green) and IFN-γ (red) expression after A/PR8 restimulation. NKp46 and IFN-γ colocalization is shown in yellow. (**C**) Representative confocal micrographs of unstimulated control or 18-hour A/PR8-restimulated PBMCs (subjects #9, #10; 1 month post-vaccination) were stained with NKp46 (green) and IFN-γ (red). Red lines indicate cell diameters. (**D**) Diameter and surface area of NKp46^+^IFN-γ^+^ (red) and NKp46^+^IFN-γ^−^ (blue) cells (n = 45). Mean ± SEM. ***P < 0.005; Student’s *t*-test. For all micrographs: scale bars, 5 μm; original magnification, 63× oil.

Confocal microscopy was then used to reveal the relationship between intracellular NKp46 and IFN-γ recall responses in NK cells. While naive NK cells mainly expressed surface NKp46 before vaccination, NK cells purified 1 month post-vaccination exhibited decreased surface NKp46 but increased intracellular NKp46 (Fig. 4A, Fig. 5A and B), likely from internalization. Thus, these post-vaccination NK cells represented potential memory-like cells. Besides phenotypic differences, these potential memory NK cells produced effector cytokines in response to A/PR8, as activated NK cells expressing some intracellular NKp46 produced IFN-γ upon A/PR8 restimulation (Fig. 5B and C).

Since NK cells became large and granular upon activation [32,33], we hypothesized that A/PR8 restimulation might change NK morphology. Expectedly, A/PR8-stimulated NKp46^+^ cells exhibited larger diameters and surface area than controls (*P* < 0.005, Fig. 5D). Above all, these larger NKp46^+^ cells were mainly IFN-γ^+^, representing cells that were able to rapidly respond after viral restimulation.

Thus, vaccination-induced receptor internalization might lead to NKp46 downregulation, and increased intracellular NKp46 expression might be a mechanism underlying the enhanced NK cell—mediated IFN-γ responses against A/PR8.

### NK cell recall response is dependent on NKp46 and specific to the influenza virus

**Fig 6:**
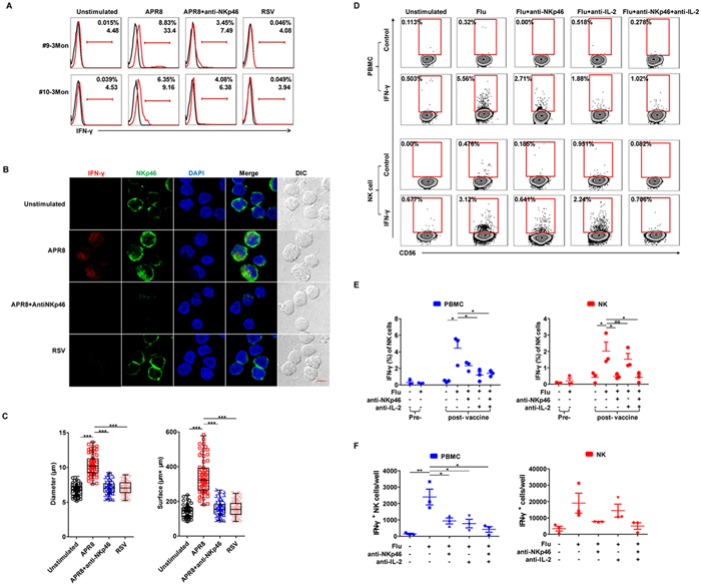
NK cell recall response is influenza-specific and blocked by anti-NKp46. PBMCs 3 months post-vaccination (subjects #9, #10) were unstimulated or stimulated with A/PR8 (2000 HA/mL) in the presence of 10 μg/mL anti-NKp46, or with RSV (MOI:1) for 18 hours. (**A**) Histograms depict IFN-γ expression (red) or isotype controls (black) on CD3^−^CD56^+^ NK cells by FACS. (**B**) Representative confocal micrographs of PBMCs (subject #10; 3 months) stained for DAPI (nuclei; blue), NKp46 (green) and IFN-γ (red) expression; colocalization is shown in yellow. Scale bar, 5 μm; original magnification: 63× oil. (**C**) Diameter and surface area of human NKp46^+^ cells in (B) (n = 60). **P < 0.01 and ***P < 0.005; one-way ANOVA. (**D**) PBMCs and purified NK cells at 2 months post-vaccination (subjects #11–13) were restimulated for 18 hours with FluaRIX vaccine (2013–2014) in the presence of 10 μg/mL anti-NKp46 and/or 4 μg/mL neutralizing rat anti—human IL-2 antibodies. Intracellular IFN-γ of CD3^−^CD56^+^ human NK cells was detected by FACS. Flu: FluaRIX. (**E**) Intracellular IFN-γ expression on NK cells detected pre-vaccination (day 0) and at 2 months post-vaccination (subjects #11–13) (PBMCs: blue bars; purified NK cells: red bars). (**F**) Absolute number of IFN-γ^+^ cells detected under corresponding conditions at 2 months post-vaccination (PBMCs: blue bars; purified NK cells: red bars) (n = 3). *P < 0.05; Student’s *t*-test.

To further investigate the mechanism underlying NK-mediated IFN-γ responses, we addressed the following 2 points: whether NKp46 was directly involved in responding to influenza antigens and whether vaccine-induced memory NK cells specifically responded to influenza antigens or also to those in other viruses, such as RSV [34]. We restimulated fresh PBMCs (subjects #9, #10) 3 months post-vaccination with A/PR8 with or without anti-NKp46 blocking antibody, or with RSV alone. While A/PR8 induced higher IFN-γ during recall compared to unstimulated cells, RSV did not; moreover, anti-NKp46 dramatically inhibited IFN-γ in both donors (Fig. 6A). These results suggested that post-vaccination NK cells produced influenza-specific IFN-γ responses and that surface NKp46 mediated this recall response, likely by recognizing HA. By confocal imaging, we found that IFN-γ and intracellular NKp46 obviously colocalized together in NK cells after A/PR8 restimulation, but not after RSV stimulation or in the absence of NKp46 signaling (Fig. 6B). Furthermore, cell diameters and surface area were significantly different among each group, where NKp46^+^ cells became larger with more cytoplasm after A/PR8 stimulation in contrast to the relatively small NKp46^+^ cells observed after anti-NKp46 treatment or RSV stimulation (Fig. 6C).

In previous work by the Greenberg group [35], IFN-γ production from NK cells was found to be dependent on IL-2 production from influenza A virus—specific T cells upon ex vivo restimulation with the influenza A antigen. To determine whether IL-2 also played a role in the NK cell recall response from recently vaccinated subjects, whole PBMCs or purified human NK cells pre- and post-vaccination were restimulated with the influenza vaccine FluaRIX in the presence of the IL-2–neutralizing antibody or the anti-NKp46 antibody. As shown in Fig. 6D, we found that purified NK cells also produced IFN-γ upon restimulation, although at lower levels than from whole PBMCs. While adding in either an anti-NKp46 antibody or an IL-2–neutralizing antibody partially decreased the NK cell production of IFN-γ in whole PBMC cultures, notably only anti-NKp46 treatment and not anti—IL-2 treatment decreased the percentage and number of IFN-γ^+^ cells from the purified NK cell cultures (Fig. 6E and F). These data indicated that NK memory recall responses were dependent on NKp46 and specific to influenza antigens, while IL-2 signals worked cooperatively with the HA—NKp46 interaction to enlarge the recall response. Overall, our data suggest that recall antigen stimulation induces both functional and morphological changes to vaccine-induced, intracellular-NKp46–expressing memory NK cells.

## Discussion

Humans often catch the flu more than once but do not always seem to benefit from a memory immune response [5]. A recent study found that the most potent CD8 T cell—inducing influenza vaccine did not induce sufficient numbers of cross-reactive CD8 T cells to provide substantial protection against a lethal non-homologous influenza A virus challenge [4]. The protection through the humoral arm of the immune response has limitations, as a seasonal influenza vaccine likely provides protection only to antigens contained in that vaccine; however, once the vaccinated host encounters a different influenza virus—they are known to rapidly mutate—the ability of the humoral response to protect the host decreases because of low HA-specific antibody titers to a heterologous influenza virus (Fig. 1F). A recent study even demonstrated that interaction between the B cell—expressed B cell receptor (BCR) with HA disrupted antibody secretion and caused influenza-specific B-cell death [36], further limiting the specific antibody responses against the influenza virus. Furthermore, peripheral NK cells, but not CD3^+^ T cells, robustly increased recall IFN-γ responses for 6 months after vaccination. In contrast to the elicited antibody response, which was effective to antigens within the vaccine strain but afforded little protection against heterologous strains like the A/PR8 virus, we found that NK cells were able to elicit IFN-γ upon restimulation by both homologous and heterologous influenza subtypes (Fig. 1E). This finding suggests that an advantage of the NK cell memory-like response may be its ability to provide broad immunity against many influenza subtypes. Further work needs to be done to assess how much this short-term memory NK cell recall response contributes to the overall protection from influenza infection after vaccination. Nonetheless, NK cells may play a role in modifying or enhancing protective cell function, perhaps similar to something like a cell-based adjuvant.

Another important finding here is that surface NKp46 expression significantly decreases while intracellular NKp46 expression increases on NK cells after vaccination. Furthermore, intracellular NKp46 expression positively correlates with IFN-γ responses to A/PR8 restimulation. This phenomenon interested us because NKp46 directly recognizes influenza HA and is implicated in human NK natural cytotoxicity and activation [17,19,37]. Our kinetic study showed profound enhancement of influenza-specific NKp46^+^ NK cell responses, a finding similar to that of Ly49H^+^ NK responses to MCMV [13] in which NK cells were previously shown to undergo virus-specific expansion through the Ly49H—MCMV m157 interaction.

In a previous study, influenza vaccination did not alter NKp46 expression [9]. This inconsistency with our results may be attributed to their relatively shorter 7- to 40-day monitoring period post-vaccination, when NKp46 expression varied only slightly in some subjects analyzed here. NK cells internalize HA following specific recognition by NKp46 and colocalize to MHC class II peptide-loading compartments [30]. We hypothesized that a similar mechanism might be working in our model. Consistent with our expectations, intracellular NKp46 was dynamically expressed post-vaccination. Intriguingly, the emergence of peripheral NKp46(I)^+^ NK cells and IFN-γ responses peaked simultaneously post-vaccination, suggesting that intracellular NKp46 positively regulated NK cell function. Notably, NKp46(I)^+^ NK cells declined to normal levels 6 months post-vaccination in some subjects. Therefore, intracellular NKp46 induction might be a limiting factor for NK responses. NKp46(I)^+^ NK frequency varied among the subjects, implying that viral-antigen experience in the host influenced NKp46 expression. Influenza infection may therefore alter the NKp46(I)^+^ NK cell repertoire and imprint memory unto NK cells; alternatively, influenza virus entry and infection of NK cells may impair and inhibit NK cells [38–40]. Thus, induction of one of two different pathways downstream of NKp46 may tip the balance toward either activating or inhibiting NK cell function.

The changes in NKp46 occur gradually over months in our model, and this observation differs from previous studies pointing to a quick, transient drop in NKp46^+^ NK cells. In particular, our study likely differed from the 2004 Hanna J et al. study [30] because they observed the more immediate rapid responses after stimulation than we evaluated here (0–30 minutes vs. months, respectively); additionally, their internalization assays were performed in vitro, which might have elicited quick responses, whereas the internalization process that we observed might have more complex kinetics after being exposed to many different in vivo external factors that could regulate this expression after vaccination. In another study by Jost S et al. in 2011, the data showed a very early (1 day after vaccination) transient decrease in the proportion of NKp46^+^ NK cells [31]. We speculate that their observed transient differences in NKp46 expression on CD56^dim^ cells may represent an initial, direct effect of influenza vaccination on these NK cells due to only the HA—NKp46 interaction, whereas the differences that we observe here at the much later time points (months) post-vaccination may be the result of a myriad of in vivo factors released during the ensuing immune response to vaccination—such as the effect of cytokines or other mediators produced by other cells after vaccination on CD56^dim^ cells—that coordinate to cause a second wave of NKp46 downregulation.

How surface NKp46 receptor internalizes into the cell and how NK cells interact with influenza during the recall phase remains to be established. The W32R mutation within the natural cytotoxicity receptor (NCR) gene in Noé mice abolished surface NKp46 expression but induced hyperresponsive NK cells [41], and silencing the *Helios* transcription factor gene regulated NKp46 expression and IFN-γ responses during NK cell development. Accordingly, identifying pathways promoting the development of NKp46(I)^+^ NK cells will be important for understanding the molecular mechanisms underlying the generation of memory NK cells and developing NK cell—based vaccines.

Since previous studies by the Greenberg and the Riley groups showed that IL-2 from influenza A—stimulated T cells was required to elicit IFN-γ from NK cells [35,42,43], we tested whether IL-2 was necessary for IFN-γ production. We treated the FluaRIX vaccine—stimulated PBMCs or purified NK cell cultures (where FluaRIX was used as the homologous antigen) with an IL-2–neutralizing antibody and showed that neutralizing IL-2 did not significantly inhibit IFN-γ production from the purified NK cells in response to the recall antigen (Fig. 6D-F). This finding is seemingly somewhat different than the findings from the Greenberg group [35], but they performed their experiments using subjects without recent flu-like symptoms rather than a population that received vaccination. In this case, their NK cell IFN-γ responses would likely be similar to our unvaccinated controls (who likely had been infected with the influenza virus in the past but did not have the short-lived memory NK cell recall responses) due to being outside of the 2 to 3 month window after vaccination when these memory NK cell recall responses were the strongest. In fact, they also found that the IL-2–neutralizing Ab only partially blocked IFN-γ production by NK cells, perhaps suggesting that other molecules like NKp46 might also be involved in the IFN-γ response of NK cells.

Regarding the role of NKp46 in the NK cell IFN-γ response, we found here that adding anti-NKp46 partially decreased the NK cell response in whole PBMC cultures as well as from purified NK cells, which is in contrast to the results observed using the IL-12–neutralizing antibody. However, because this experiment most likely blocked surface, but not internal, NKp46 (Fig. 6A), there were certain limitations to the interpretation of our results. Our results most likely suggest the importance of NKp46 binding to the virus during the recall response. We speculate that perhaps some surface NKp46 expression is required to initiate signals after HA interaction via the ITAMs on the NKp46-associated FcRs that lead to IFN-γ production, and intracellular NKp46 then acts to amplify these signals; this potential mechanism involving the intracellular NKp46 in memory function remains to be further investigated in future studies.

A recent study demonstrated that the costimulatory molecule DNAM-1 (CD226) is dynamically regulated on Ly49H^+^ NK cells and required for differentiation of memory NK cells during MCMV infection[14]. Further investigation into the roles of costimulatory molecules like CD226 and costimulatory cytokine signaling as well as the mechanism underlying IFN-γ production (including the roles of the related transcription factors Helios and T-bet) will be important for understanding the molecular mechanisms underlying the generation of memory NK cells, providing potential insights into the biology of human NK cells for vaccine development and NK cell—relevant diseases.

## Supporting Information

S1 Fig Schematic representation of the secondary human NK cell immune response following influenza virus vaccination. **(A)** Twenty-seven healthy adult volunteers between 20 to 47 years old that never previously received influenza vaccine were enrolled, and their plasma influenza antibody IgM levels were measured by ELISA. (**B**) Thirteen IgM-negative volunteers were selected and divided into 2 groups: 11 volunteers received the inactivated split influenza vaccine composition by intramuscular (i.m.) injection, and 2 controls were not inoculated. PBMCs were isolated from the subjects beginning at day 0 before vaccination to ~6 months after vaccination at the indicated time points. NK cell phenotypes and HA-specific antibody titers to influenza virus were evaluated. PBMCs and purified NK cells were also cultured in vitro with heterologous influenza virus A (PR8 strain) or the corresponding homologous FluaRIX vaccines to evaluate NK cell recall responses.
(TIF)

S2 Fig NKG2C and CD57 expression on CD3^−^CD56^+^ NK cells remained relatively stable. PBMCs from vaccinated subjects (#9, #10) were analyzed by flow cytometry for CD57 and NKG2C expression on gated CD3^−^CD56^+^ NK cells at the indicated time points following vaccination.
(TIF)

S3 Fig FACS gating strategy for surface and intracellular NKp46^+^ expression on NK cells. FACS gating strategy for surface and intracellular NKp46 expression on CD3^−^CD56^dim^ NK cells within the lymphocyte gate. PBMCs were isolated from vaccinated subjects (#10) on day 0.
(TIF)

S1 Table Demographic information on the human volunteers. Detailed demographic information regarding the human volunteers used in our study, including age and sex.
(DOC)
