# Supplementary figures and images for: Influenza Vaccine Induces Intracellular Immune Memory of Human NK Cells
Source: PLoS One. 2015 Mar 17;10(3):e0121258. doi: 10.1371/journal.pone.0121258 (PMC4363902; doi:10.1371/journal.pone.0121258)

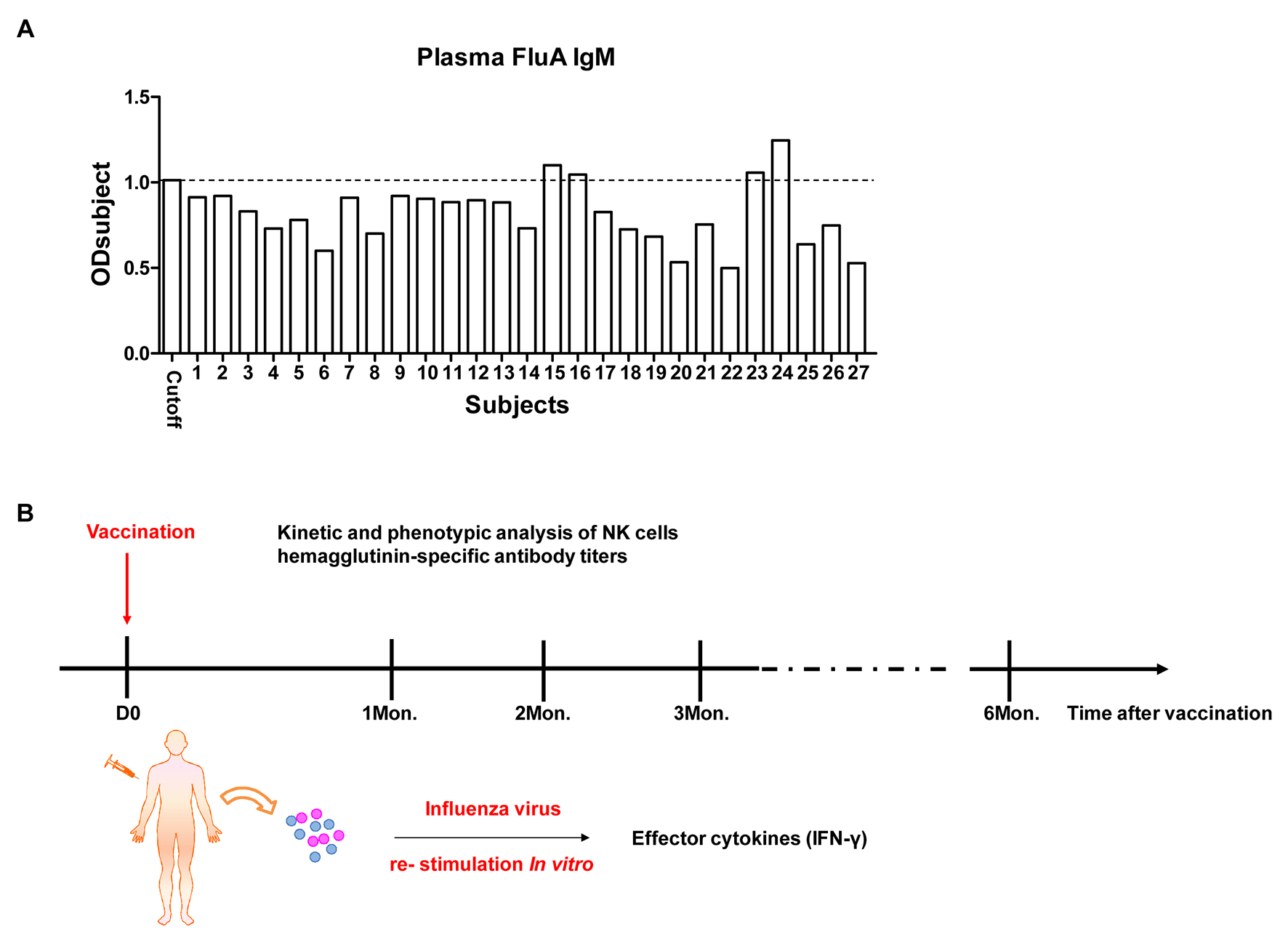

Supplement: S1 Fig — (A) Twenty-seven healthy adult volunteers between 20 to 47 years old that never previously received influenza vaccine were enrolled, and their plasma influenza antibody IgM levels were measured by ELISA. (B) Thirteen IgM-negative volunteers were selected and divided into 2 groups: 11 volunteers received the inactivated split influenza vaccine composition by intramuscular (i.m.) injection, and 2 controls were not inoculated. PBMCs were isolated from the subjects beginning at day 0 before vaccination to ~6 months after vaccination at the indicated time points. NK cell phenotypes and HA-specific antibody titers to influenza virus were evaluated. PBMCs and purified NK cells were also cultured in vitro with heterologous influenza virus A (PR8 strain) or the corresponding homologous FluaRIX vaccines to evaluate NK cell recall responses. (TIF) [file pone.0121258.s001.tif]

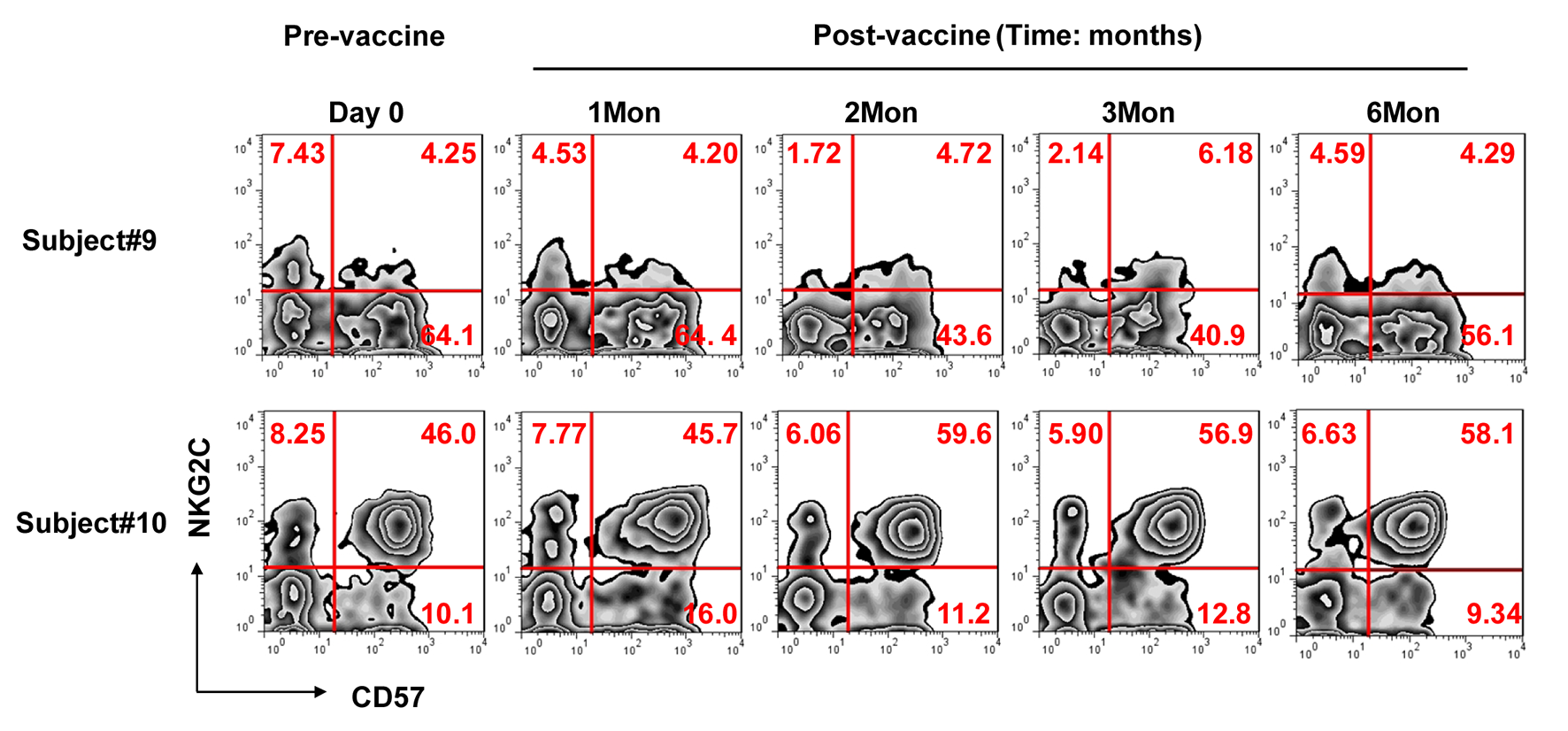

Supplement: S2 Fig — PBMCs from vaccinated subjects (#9, #10) were analyzed by flow cytometry for CD57 and NKG2C expression on gated CD3−CD56+ NK cells at the indicated time points following vaccination. (TIF) [file pone.0121258.s002.tif]

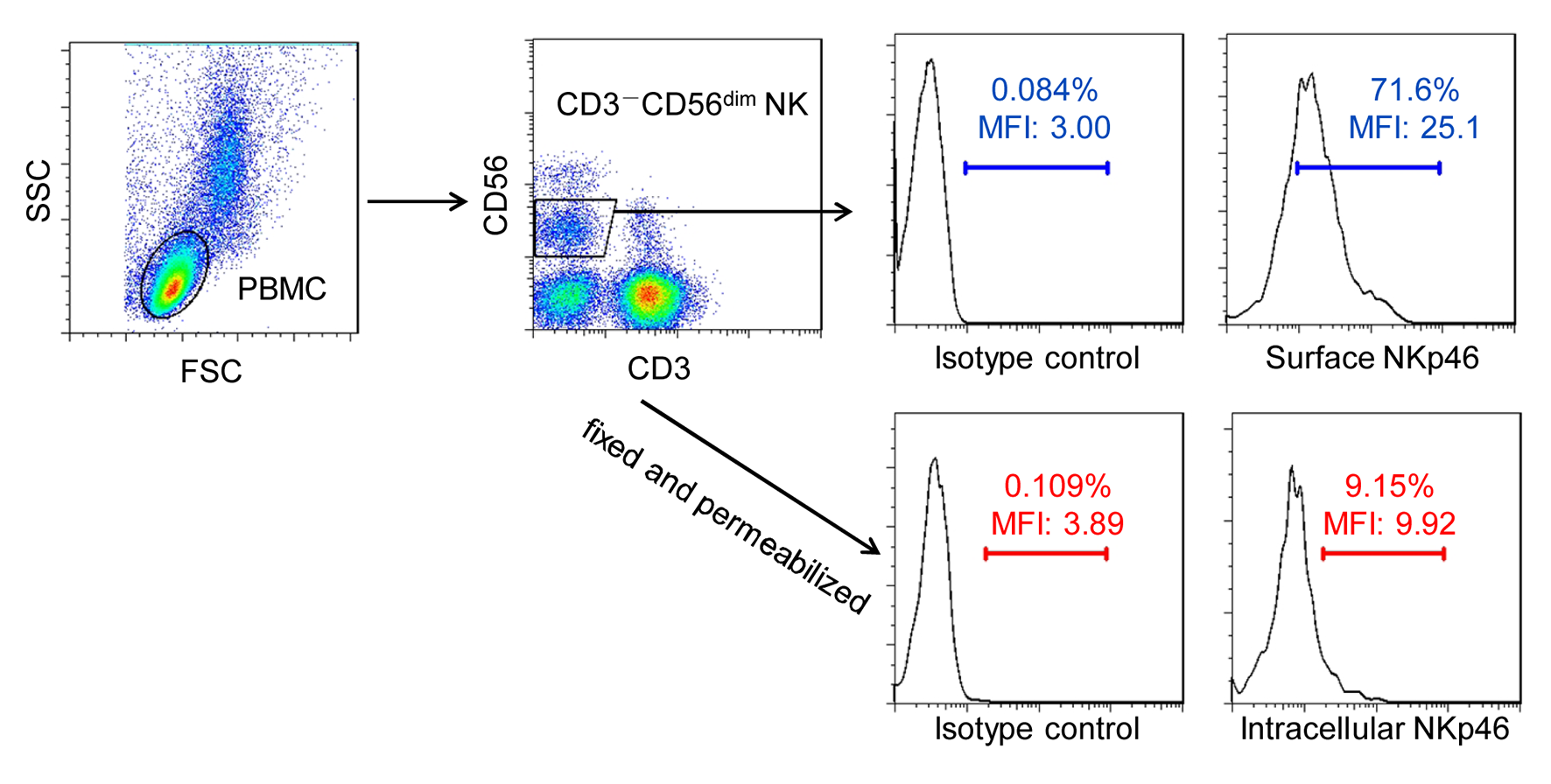

Supplement: S3 Fig — FACS gating strategy for surface and intracellular NKp46 expression on CD3−CD56dim NK cells within the lymphocyte gate. PBMCs were isolated from vaccinated subjects (#10) on day 0. (TIF) [file pone.0121258.s003.tif]
