# Supplementary material for: Influenza Vaccine Induces Intracellular Immune Memory of Human NK Cells
Source: PLoS One. 2015 Mar 17;10(3):e0121258. doi: 10.1371/journal.pone.0121258 (PMC4363902; doi:10.1371/journal.pone.0121258)
Supplement: S1 Table — Detailed demographic information regarding the human volunteers used in our study, including age and sex. (DOC) [file pone.0121258.s004.doc]

**S1 Table.** Demographic information on the human volunteers

| **Subject** | **Age (years)** | **Sex** |
| --- | --- | --- |
| #1 | 21 | Female |
| #2 | 22 | Male |
| #3 | 25 | Female |
| #4 | 28 | Male |
| #5 | 23 | Male |
| #6 | 24 | Female |
| #7 | 22 | Female |
| #8 | 22 | Male |
| #9 | 24 | Male |
| #10 | 26 | Female |
| #11 | 24 | Male |
| #12 | 24 | Male |
| #13 | 28 | Female |
| #14 | 27 | Female |
| #15 | 26 | Male |
| #16 | 32 | Female |
| #17 | 24 | Male |
| #18 | 26 | Female |
| #19 | 23 | Male |
| #20 | 21 | Female |
| #21 | 32 | Female |
| #22 | 30 | Male |
| #23 | 20 | Male |
| #24 | 25 | Female |
| #25 | 30 | Male |
| #26 | 47 | Male |
| #27 | 24 | Female |
| Mean (year) | 26 | —— |
